# Serial urinary neutrophil gelatinase associated lipocalin in pediatric diabetic ketoacidosis with acute kidney injury

**DOI:** 10.1186/s40842-021-00133-8

**Published:** 2021-11-01

**Authors:** Vijai Williams, Muralidharan Jayashree, Karthi Nallasamy, Devi Dayal, Amit Rawat, Savita Verma Attri

**Affiliations:** 1 Division of Pediatric Intensive Care, Department of Critical Care, Sheikh Khalifa Medical City (SKMC), Abu Dhabi, United Arab Emirates; 2 Division of Pediatric Emergency and Intensive Care, Department of Pediatrics, Advanced Pediatrics Centre, Post Graduate Institute of Medical Education & Research, Chandigarh, India; 3 Division of Pediatric Endocrinology, Department of Pediatrics, Advanced Pediatrics Centre, Post Graduate Institute of Medical Education & Research, Chandigarh, India; 4 Division of Pediatric Allergy and Immunology, Department of Pediatrics, Advanced Pediatrics Centre, Postgraduate Institute of Medical Education and Research, Chandigarh, India; 5 Division of Pediatric Biochemistry, Department of Pediatrics, Advanced Pediatrics Centre, Postgraduate Institute of Medical Education and Research, Chandigarh, India

**Keywords:** Type1 diabetes, Ketoacidosis, Pediatric, Acute kidney injury, Lipocalin

## Abstract

**Background:**

Acute kidney injury (AKI) due to Diabetic Ketoacidosis (DKA) is rather common. Novel biomarkers to diagnose AKI are being increasingly used in different settings. The use of urinary Neutrophil Gelatinase-Associated Lipocalin (uNGAL) in predicting persistent AKI in pediatric DKA cases is still not thoroughly investigated.

**Methods:**

This was a secondary analysis of Saline versus Plasma-Lyte in Ketoacidosis (SPinK) trial data; 66 children (> 1 month-12 years) with DKA, defined by the International Society for Pediatric and Adolescent Diabetes (ISPAD), were analyzed. Children with cerebral edema, chronic kidney disease and those who received pre-referral fluids and/or insulin were excluded. uNGAL and urine NGAL-creatinine ratio (uNCR) at 0 and 24 h were measured in all. Persistent AKI was defined as a composite outcome of continuance of AKI defined by the Kidney Disease Improving Global Outcomes (KDIGO) stage 2 or 3 beyond 48 h from AKI onset, progression of AKI from either KDIGO stage 0 or 1 to a worse stage, need of renal replacement therapy or death.

**Main outcomes:**

Thirty-five (53%) children had AKI at admission; 32 (91.4%) resolved within 48 h. uNGAL was significantly higher in the AKI group at admission [79.8 ± 27.2 vs 54.6 ± 22.0, p = 0.0002] and at 24 h [61.4 ± 28.3 vs 20.2 ± 14.5, p = 0.0003]. Similar trend was observed with uNCR at admission [6.7 ± 3.7 vs 4.1 ± 2.6, p = 0.002] and at 24 h [6.3 ± 2.5 vs 1.2 ± 1.0, p = 0.01]. Furthermore, uNGAL at admission showed a moderate positive linear correlation with serum creatinine. Additionally, elevated uNGAL at 0 and 24 h correlated with corresponding KDIGO stages. Admission uNGAL >88 ng/ml and uNCR of >11.3 ng/mg had a sensitivity of 66% and 67%, specificity of 76% and 95%, and Area under the receiver operating characteristic curve (AUC) of 0.78 and 0.89 respectively for predicting persistent AKI at 48 h.

**Conclusions:**

Majority of AKI resolved with fluid therapy. While uNGAL and uNCR both correlated with serum creatinine and AKI stages, serial uNCR was a better predictor of persistent AKI than uNGAL alone. However, feasibility of routine uNGAL measurement to predict persistent AKI in DKA needs further elucidation.

**Trial registration:**

This was a secondary analysis of the data of SPinK trial [CTRI/2018/05/014042 (ctri.nic.in)].

**Supplementary Information:**

The online version contains supplementary material available at 10.1186/s40842-021-00133-8.

## Background

Diabetic ketoacidosis (DKA) accounts for almost a quarter of all diabetes-related admissions to a hospital [1, 2]. Several factors, including duration of illness, delayed healthcare seeking, severity of DKA, associated comorbidities, choice of fluid type and volume, and the extent of organ dysfunction determine outcome [3–5]. Among organ dysfunctions, acute kidney injury (AKI) has been reported in about 30–65% of children with DKA, at the time of presentation [6–9].

AKI in DKA, is largely pre-renal and resolves with adequate hydration [10–12]. However, it can progress to acute tubular injury if acidosis is severe and prolonged, or when DKA is associated with sepsis*.* Furthermore, compromised renal blood flow and oxidative stress may cascade into endothelial dysfunction, coagulopathy and mitochondrial injury, resulting in intrinsic AKI [13, 14]. In a few children, AKI can progress to chronic kidney disease (CKD) [14, 15]. Chen et al. found an association between severity of AKI and risk of rapid progression of CKD in patients hospitalized with DKA [15]. Thus, the development of AKI in DKA has a bearing on both, short-term outcomes like need of renal replacement therapy (RRT), length of hospital stay and mortality, as well as long term outcomes like progression to CKD and increase in cost of care. Early recognition of children in whom AKI is likely to persist may help in prognostication.

AKI may occur in critically ill children in a pediatric intensive care unit (PICU) due to a number of etiologies. From a clinician’s perspective, however, it is important to differentiate transient AKI that would respond to fluid therapy, from persistent AKI (as defined by the Sixteenth Consensus Conference of the Acute Dialysis Quality Initiative (ADQI-16) that may require additional renal support. The most widely used marker to detect occurrence and predict persistence of AKI is the rise and persistent elevation of serum creatinine [10, 11]. However, it has a delayed rise after AKI and can be unreliable, as it is often altered by several non-renal factors such as age, body weight, muscle mass, liver dysfunction and protein intake [16]. Similarly, the use of estimated glomerular filtration rate (GFR) as a marker for improvement or worsening of AKI may be imprecise in a setting of dehydration. To address these shortcomings, novel biomarkers are being increasingly used both, for early detection, as well as prediction of non-resolution of AKI.

Urinary neutrophil gelatinase-associated lipocalin (uNGAL) has being studied in different clinical situations as a potential biomarker for early detection of AKI [17]. This 25 kDa protein, that was found first in renal tubules, gets upregulated in epithelial injury due to ischemia [18–20], contrast [21], post cardiac surgery [17, 22, 23] or post-transplantation [24–26]. The utility of serum and urinary NGAL to discriminate transient from persistent AKI, however, is limited to very few studies in critically ill adults [27, 28], and the data in critically ill children is scarce [29].

In our setting, children with DKA tend to present late, with greater severity of acidosis and in sepsis or shock; these are all factors that lead to persistent AKI. We hypothesized that serial uNGAL measurements are better than serum creatinine alone in differentiating persistent from transient AKI in children with DKA.

## Materials and methods

### Study design and setting

This was a secondary analysis of the data collected prospectively during the conduct of Saline versus Plasma-Lyte in Ketoacidosis (SPinK) Trial [12]- a single center randomized controlled trial from August 2017 to December 2018 in the Pediatric Emergency and Intensive Care Units of a large tertiary, teaching and referral hospital in India. The objective was to determine the relationship between uNGAL and severity of AKI associated with pediatric DKA and to determine the usefulness of uNGAL in distinguishing transient from persistent AKI.

### Definitions

#### Acute kidney injury

AKI was defined as per Kidney Disease: Improving Global Outcomes (KDIGO) criteria [30]. As pre-admission/ baseline serum creatinine was not available, bedside Schwartz formula was used for its estimation, assuming GFR of 127 mL/min for children above 1 year of age and 103 mL/min for those below 1 year [11, 31].

#### Transient AKI

Resolution of AKI was defined as the return of serum creatinine from KDIGO stage 2 or 3 (classified as AKI) to either KDIGO stage 0 or 1 (classified as non- AKI) within 48 h of therapy. The rationale for using this 48-h cut-off was two-fold; firstly, most children recover from AKI within 48 h and secondly, children who may need additional intervention may be identified early [11, 32, 33].

#### Persistent AKI

Persistent AKI was defined as a composite outcome of, continuance of AKI (KDIGO stage 2 or 3) beyond 48 h from AKI onset, progression of AKI from either KDIGO stage 0 or 1 to a worse stage, need of RRT or death.

### Participants

We enrolled all children, aged > 1 month to < 12 years, with a diagnosis of DKA, as defined by the International Society for Pediatric and Adolescent Diabetes (ISPAD) 2014 (pH < 7.3, Bicarbonate < 15 mEq/L and Ketones > 3mmmol/l) [34] presenting to our pediatric Emergency Department (ED). Children with Glasgow Coma Scale (GCS) < 8 at presentation, previously diagnosed chronic kidney disease or liver disease and those who received pre-referral fluids and/or insulin at hospital presentation were excluded. Severity of DKA was classified as mild, moderate and severe if the pH was between 7.2 and 7.3, between 7.1 and 7.2 and < 7.1 respectively as per the ISPAD guidelines.

### Treatment protocol and monitoring

Eligible children, if in shock, received isotonic fluid bolus of 20 ml/kg over an hour, else the fluid volume was calculated as a sum of deficit (65-85 ml/kg) and maintenance for the next 48 h and administered as an hourly infusion. Insulin was started at 0.05 U/kg/hour in all, after an initial hour of fluid therapy. Dextrose was added once blood glucose fell below 250 mg/dl. In case of persistent hyperglycemia, the clinician went through a checklist including patency of intravenous access, insulin preparation and its shelf life, and appropriateness of dilution before increasing insulin to 0.1 U/Kg/hour. The protocol was followed till resolution of DKA defined by pH > 7.3, bicarbonate > 15 mEq/L & normal sensorium.

### Data collection

Clinical data (respiratory rate, pulse rate, capillary refill time, blood pressure, fluid intake and urine output) were continuously recorded and the values were entered in a pre-designed monitoring sheet. The need for RRT was assessed daily. In addition to renal failure-related data points, the duration of mechanical ventilation (MV), length of ICU and hospital stay, and in hospital mortality were recorded.

### Laboratory investigations

Blood glucose (capillary or venous) was checked hourly, while blood gas analysis was done every 4 h. Urea, creatinine and electrolytes were measured 4–8 hourly for a minimum of 48 h or until resolution of AKI. Urine samples were collected at admission, concurrent with urinary catheterization and then at 24 h after the initiation of fluids. Spot uNGAL and corresponding urine creatinine were measured at admission and at 24 h. Spot uNGAL to urine creatinine ratio (uNCR) was obtained by dividing spot uNGAL and corresponding urine creatinine. This was done to mitigate the effect of glomerular filtration on the level of biomarker. All children underwent a urine microscopy and culture and if positive, were excluded from analysis as urinary tract infection (UTI) can erroneously increase uNGAL values.

### Test methods

Urine was centrifuged (at 2000 rpm for 15 min at room temperature) to remove cellular debris. The clear supernatant was stored at − 80 °C until measurement. The concentration of uNGAL was estimated with the commercial NGAL ELISA kit (Bio Vendor Human Lipocalin-2/NGAL ELISA), in a sandwich enzyme immunoassay. Limit of NGAL detection was 0.02 ng/ml with inter-assay CV of 7.8% and intra-assay coefficient of variance (CV) of 9.7–9.8%.

### Statistical analysis

Data is represented as mean with standard deviation or median with inter-quartile range as per normality of distribution. Unpaired Students’ t- test or Wilcoxon rank-sum test were used for intergroup comparisons of parametric data and Mann Whitney test or Fischer Exact test for non-parametric data. Unadjusted Chi-Square test was used to analyze the differences in categorical outcome. Patients were divided to two groups viz., AKI and Non-AKI based on their KDIGO staging at admission. The degree of uNGAL elevation was compared with respect to AKI stage at admission and at 24 h. Correlation between uNGAL and creatinine elevation was done. Receiver operating characteristics (ROC) curve was generated and area under curve (AUC) was calculated to assess the discriminatory power of uNGAL to predict persistent AKI at 48 h. The optimal cut off points for sensitivity and specificity were determined by the largest sum of sensitivity and specificity. We compared the absolute urinary biomarker levels (uNGAL and uNCR) at admission along with their respective percentage decline over next 24 h in predicting persistent AKI at 48 h. A *p*-value (2 tailed) < 0.05 was considered significant in all analyses. Analyses were performed using SPSS software version 23 (IBM SPSS Statistics for Windows, Version 23.0, Armonk, NY, USA).

## Results

### Flow of patients

Seventy-seven eligible children were admitted to ED. Eleven were excluded: 8 had received insulin and fluids prior to admission, 2 had cerebral edema and 1 child with mild DKA denied consent. None had chronic kidney disease or liver dysfunction during screening. At the end of screening, 66 children were included in the study. Baseline characteristics of study subjects are as shown in Table 1.

**Table 1:** Baseline characteristics

Characteristics	n = 66
Age, years	7.2 ± 3.8
Male gender, n (%)	33 (50)
Weight, kg	19.2 ± 9.2
Height, cm	112.9 ± 22.3
Body Mass Index	14.2 ± 2.1
Weight (z score)	−1.90 ± 1.68
Height (z score)	− 1.36 ± 1.26
Body Mass Index (z score)	−1.79 ± 1.49
New onset DKA, n (%)	41 (62.1)
Duration of diabetes in prior diagnosed, months, Median (IQR)	17.4 (7.7, 39.9)
Duration of prodromal symptoms, days, Median (IQR)	5 (2,10)
GCS, Median(IQR)	13 (11,15)
Severity of DKA	
Mild, n%	4 (6.1)
Moderate, n%	22 (33.3)
Severe, n%	40 (60.6)
**Lab values at presentation**	
Glucose, mg/dl	487 ± 151
Blood Ketones, mmol/L	5.5 ± 0.9
pH	7.02 ± 0.15
Sodium, mmol/L	137.7 ± 6.9
Corrected sodium, mmol/L	143.5 ± 6.91
Chloride, mmol/L	111.8 ± 8.87
Potassium, mmol/L	4.02 ± 0.89
Urea, mg/dl	47.2 ± 39.5
Creatinine, mg/dl	0.84 ± 0.43
Anion gap, mmol/L	19.9 ± 5.1
Osmolality, mmol/kg	301.2 ± 15.1
Lactate, mmol/L	2.06 ± 0.88
Phosphate, mmol/L	2.7 ± 0.98
Hemoglobin, g/dl	11.5 ± 2.0
HbA_1C_,%	12.5 ± 2.4

† All values expressed as Mean ± Standard deviation

### Incidence and determinants of AKI at admission

At admission, 35 (53%) children had AKI; 24/35 (68.6%) in stage 2 and 11/35 (31.4%) in stage 3. Baseline characteristics of the 2 groups, Non-AKI and AKI (Stage 2 and 3), were comparable. The AKI group had higher mean blood glucose, higher osmolality, lower GCS and higher proportion of severe DKA (Table 2). Serum sodium (absolute and corrected for glucose), chloride and phosphate levels were similar. However, there were no differences between groups in time to endpoint of DKA and duration of PICU or hospital stay.

**Table 2:** Comparison of AKI and Non AKI groups at admission

Parameters	Total (n = 66)	AKI (*n* = 35)	Non AKI (*n* = 31)	P
Baseline Characteristics				
Age, years	7.2 ± 3.8	6.9 ± 4.0	7.5 ± 3.8	0.53
Male gender, n (%)	33 (50)	16 (45.7)	17 (54.8)	0.46
BMI	14.2 ± 2.1	14.1 ± 2.2	14.3 ± 2.1	0.75
New onset DKA, n(%)	41 (62.1)	23 (65.7)	18 (58.1)	0.06
Duration of diabetes, months, Median (IQR)	17(8, 40)	30 (8, 45)	17 (8, 33)	0.62
Duration of prodrome, days, Median (IQR)	5 (2,10)	3 (2,7)	7 (4,10)	0.09
GCS, Median(IQR)	13 (11,15)	12 (10,14)	15 (12,15)	*0.005*
Severity of DKA				
Mild and moderate, n(%)	26 (39.4)	7 (20)	19 (61.3)	*0.001*
Severe, n(%)	40 (60.6)	28 (80)	12 (38.7)	
Lab values at presentation				
Glucose at 0 h, mg/dl	487 ± 151	523 ± 156	445 ± 135	0.07
Glucose at 1 h, mg/dl	447 ± 151	476 ± 146	415 ± 106	0.09
Blood Ketones, mmol/L	5.5 ± 1.0	5.4 ± 0.9	5.6 ± 1.0	0.20
pH	7.02 ± 0.15	6.97 ± 0.12	7.08 ± 0.15	*0.002*
Bicarbonate, mmol/L	7.4 ± 2.5	6.9 ± 2.4	7.8 ± 2.5	0.06
Sodium, mmol/L	138 ± 6.9	139 ± 7.6	136 ± 6.1	0.33
Corrected sodium, mmol/L	144 ± 6.9	145 ± 7.7	142 ± 5.5	0.08
Chloride, mmol/L	112 ± 8.8	112 ± 10.5	111 ± 7.1	0.87
Potassium, mmol/L	4.0 ± 0.89	4.3 ± 0.9	3.7 ± 0.7	*0.02*
Anion gap, mmol/L	19.9 ± 5.1	20.4 ± 5.3	19.4 ± 5.0	0.51
Osmolality, mmol/kg	301 ± 15	306 ± 16	295 ± 12	*0.0001*
Phosphate, mmol/L	2.7 ± 0.98	2.7 ± 0.9	2.7 ± 1.1	0.84
HbA_1C_, %	12.5 ± 2.4	12.6 ± 2.5	12.3 ± 2.6	0.63
Fluid therapy				
Fluids received, ml/kg [Median (IQR)]	66 (46,100)	80 (55, 103)	58 (37,93)	*0.04*
Urine output, ml/kg/hr	2.9 (2.2,3.5)	2.7 (2.0,3.5)	3.0 (2.5, 3.8)	*0.54*
Creatinine, mg/dl				
0 h	0.85 ± 0.43	1.1 ± 0.41	0.55 ± 0.17	*< 0.0001*
24 h	0.56 ± 0.44	0.68 ± 0.57	0.42 ± 0.14	*0.002*
48 h	0.49 ± 0.44	0.6 ± 0.5	0.35 ± 0.11	*0.001*
uNGAL, ng/ml				
0 h	67.8 ± 27.7	79.8 ± 27.2	54.6 ± 22.0	*0.0002*
24 h	24.2 ± 20.2	61.4 ± 28.3	20.2 ± 14.5	*0.0003*
Decline over 24 h (%)	62.3 ± 25.9	21.5 ± 17.0	66.8 ± 21.7	*0.0003*
uNCR, ng/mg				
0 h	5.4 ± 3.4	6.7 ± 3.7	4.1 ± 2.6	*0.002*
24 h	2.8 ± 1.7	6.3 ± 2.5	1.2 ± 1.0	*0.01*
Decline over 24 h (%)	71.0 ± 25.5	59.8 ± 38.4	74.7 ± 16.08	*0.04*
Outcome				
Duration to achieve end points, hours	15.0 ± 7.1	16.4 ± 7.1	13.6 ± 7.0	0.11
Length of PICU stay, hours	48 ± 21	52 ± 24	42 ± 17	0.17
Length of Hospital stay, days	10 ± 4.6	10 ± 4.8	10 ± 4.4	0.93

†All values expressed as Mean ± Standard deviation

### Relationship between uNGAL and severity of AKI

**Fig. 1:**
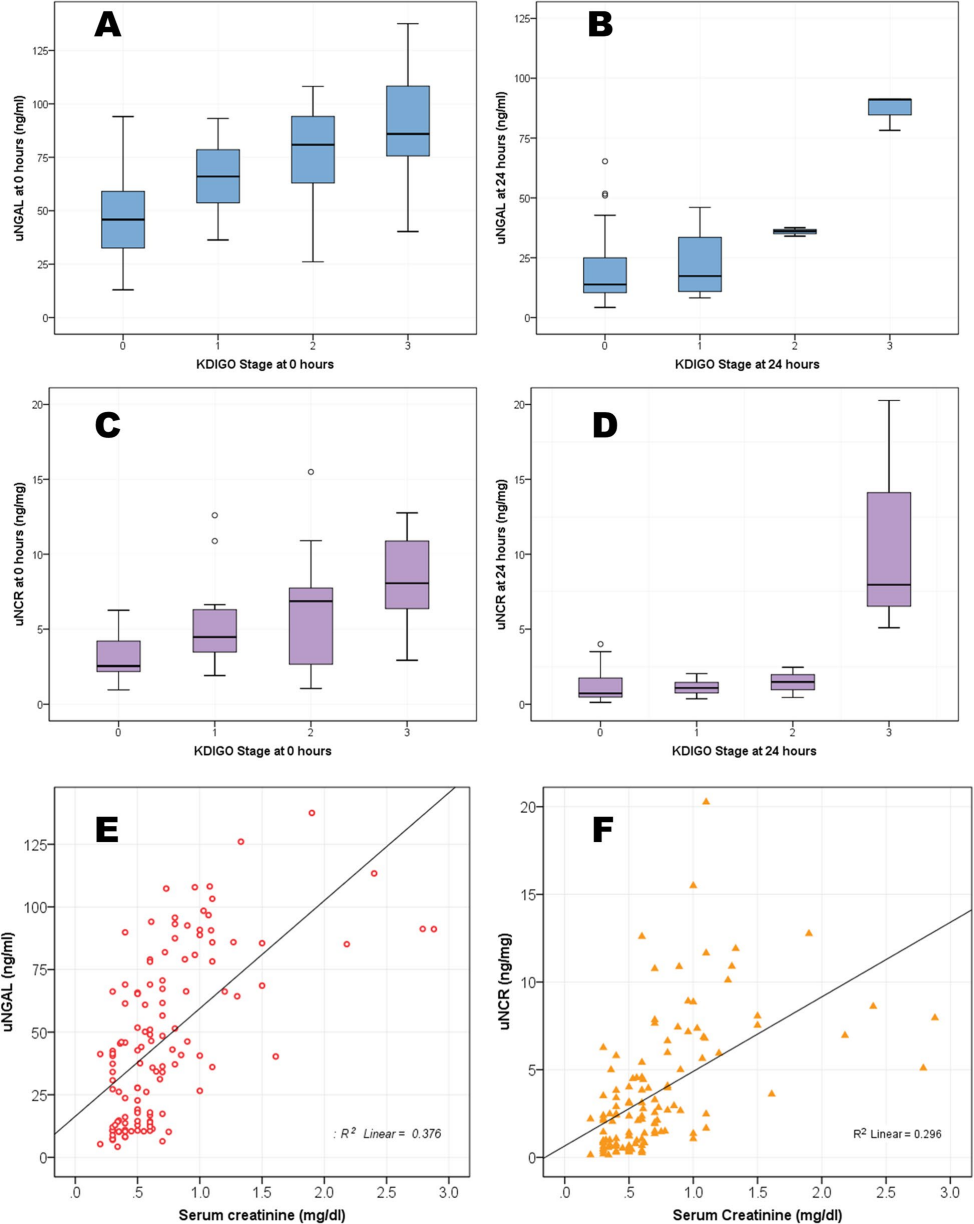
Panel A and B: Box plot representing uNGAL in relation to KDIGO Stage at 0 and 24 h respectively; Panel C and D: Box plot representing uNCR in relation to KDIGO Stage at 0 and 24 h respectively; Panel E and F: Scatter plot representing correlation of uNGAL and uNCR to creatinine

The uNGAL at admission and 24 h was significantly higher in the AKI group [79.8 ± 27.2 vs. 54.6 ± 22.0, *p* = 0.0002] and [61.4 ± 28.3 vs. 20.2 ± 14.5, *p* = 0.0003] respectively (Table 2). Similarly, uNCR was significantly higher in the AKI group, both at admission [6.7 ± 3.7 vs. 4.1 ± 2.6, *p* = 0.002] and 24 h [6.3 ± 2.5 vs. 1.2 ± 1.0, *p* = 0.01]. The elevation of uNGAL and uNCR at admission and 24 h were proportionate to the KDIGO staging at admission and 24 h respectively (Fig. 1 and Table 3). Although the absolute value of uNGAL at 24 h decreased with fluid therapy, it was commensurate with severity of AKI stage (Table 3). Meanwhile, uNGAL and uNCR showed a moderate positive linear correlation with serum creatinine; Pearson correlation coefficient of 0.61 and 0.54 respectively (Fig. 1). The percentage decline in uNGAL from admission to 24 h of fluid replacement was significantly lower in AKI compared to Non-AKI group [21.5 ± 17.0 vs. 66.8 ± 21.7, p = 0.0003]. Similarly, the percentage decline of uNCR also was lower in AKI group [59.8 ± 38.4 vs.74.7 ± 16.08, *p* = 0.04].

**Table 3:** Parameters for predicting persistent AKI at 48 h

Parameter	Value	Sensitivity	Specificity	AUC (CI)	P
uNGAL at 0 h, ng/ml	66.0	100	48	0.78 (0.65,0.92)	0.09
	83.5	100	69		
	88.1	66	76		
uNCR at 0 h, ng/mg	5.9	100	63	0.89 (0.75,1.0)	0.02
	6.9	100	73		
	11.3	67	95		
Decline of uNGAL over 24 h (%)	25	100	95	0.97 (0.93,1.0)	0.006
	50	100	82		
Decline of uNCR over 24 h (%)	50	100	93	0.98 (0.94,1.0)	0.005
	75	100	52		

†uNGAL- urinary Neutrophil gelatinase-associated lipocalin, uNCR- urinary Neutrophil gelatinase-associated lipocalin- Creatinine ratio, AKI- acute kidney injury, AUC- Area under curve

### uNGAL and prediction of persistent AKI

**Fig. 2:**
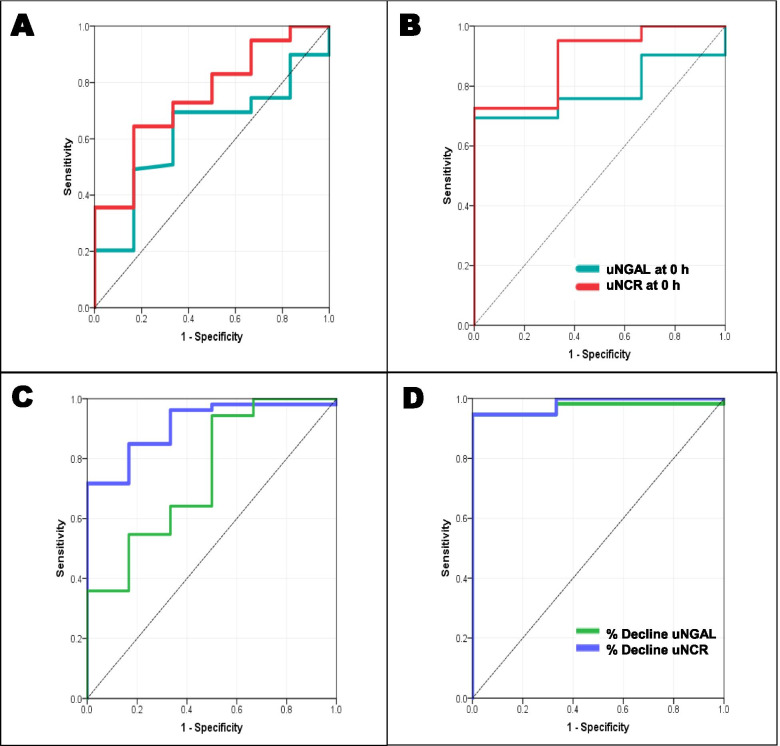
Panel A and B: ROC showing discriminating ability of uNGAL and uNCR at 0 h in resolution of AKI at 24 and 48 h respectively; Panel C and D: ROC showing discriminating ability of % decline in uNGAL and uNCR at 24 h in resolution of AKI at 24 and 48 h respectively

Of the 35 children with AKI at admission, resolution was seen in 29 (83%) at 24 h and 32 (91.4%) at 48 h following fluid correction (Additional File 1). Receiver operating characteristics (ROC) curves plotted for predictors of persistent AKI at 48 h are shown in Fig. 2. The sensitivity, specificity and AUC [95% Confidence Interval (CI)] of uNGAL and uNCR that were measured at admission to predict persistent AKI after 48 h are shown in Table 3. uNGAL > 88 ng/ml at admission had a sensitivity of 66% and specificity of 76% (PPV-11.8%, NPV-98%) while uNCR of > 11.3 ng/mg at admission, had 67% sensitivity and 95% specificity (PPV-40%, NPV-98%) in predicting persistent AKI at 48 h with AUC of 0.78 and 0.89 respectively.

Percentage decline in uNGAL and uNCR had better AUC than absolute uNGAL and uNCR values with an AUC of 0.97 and 0.98 respectively in predicting persistent AKI at 48 h (Table 3). Percentage decline in uNCR fared better than uNGAL with a < 50% fall of uNCR showing 100% sensitivity and 93% specificity, while < 50% fall of uNGAL from baseline showing an 100% sensitivity and 82% specificity in predicting AKI.

### Outcome after 28 days follow up

Of 3 children with persistent AKI at 48 h, one improved during hospital stay and 2 (3%) died; one child succumbed to fungal sepsis, shock and multi organ dysfunction at 72 h and other to progressive MODS  at 56 h of PICU stay. At 28-days follow up, all survivors had complete resolution of AKI.

## Discussion

Our study, which is novel in children with DKA, gives preliminary evidence on utility of uNGAL in AKI. AKI was present in more than half of our cohort at the time of admission with DKA; the majority (91.4%) were transient and resolved with fluids within 48 h. Persistent AKI was seen in a very few children. We found that uNGAL and uNCR elevations corresponded to AKI stage. While both were good predictors of persistent AKI, uNCR performed better than uNGAL. Serial decrease in levels over time was more prognostic than spot values.

Over the last two decades, as the understanding of AKI has evolved, the emphasis has shifted from early detection of AKI to early prediction and pattern of recovery from AKI. Kellum et al., analyzed 16,968 critically ill patients to describe 5 distinct patterns of recovery. Early reversal (*n* = 4508; 26.6%) was the most common, followed by no reversal (4496; 26.5%), early reversal with one or more relapses, but ultimate recovery (22.5%), relapsing without recovery (14.7%), and late reversal after day 7 (9.7%). Age-adjusted survival at 1 year was > 90% for early reversal and < 40% for no reversal [10]. This data reiterates the need for predicting the pattern of AKI resolution so as to target interventions and improve outcome. Transient and persistent AKI have different clinical phenotypes and outcomes. Persistent AKI was associated with worse outcomes irrespective of the degree of severity.

DKA being a state of volume depletion, the occurrence of AKI is common [6]. Adequate and timely fluid replacement mostly reverses AKI. However, in some patients, AKI tends to become intrinsic due to factors such as delayed diagnosis, prolonged uncorrected hypovolemia, severe acidosis and associated sepsis [3, 4, 7]. In such a setting, over-reliance on creatinine to monitor progression or recovery can lead to a false sense of complacency. Large volume resuscitation may decrease creatinine secondary to dilution or increased creatinine filtration [35]. Given these caveats, early detection of progressive AKI calls for a non-creatinine-based marker. This has been emphasized by the ADQI-16 to identify patients at high risk for persistent AKI.

In this context, we believe our findings that show good correlation between uNGAL and uNCR and elevated serum creatinine hold promise. uNGAL and uNCR were discriminative of KDIGO stages both at 0 and 24 h of admission. The degree of elevation was proportionate to the KGIDO stage of AKI. Such correlation has been consistently reported in other studies as well [36–38]. While interpreting uNGAL values, age-specific variations need to be kept in mind. Infants tend to have a higher uNGAL, which decreases with age. Previously published uNGAL values, range from 1.64 (0.25–5.77) ng/mL in healthy children [39] to 5 (2–150) ng/mL in very low birthweight infants [40]. Rybi-Szumińska et al., measured uNCR in 172 healthy children and adolescents and found a similar decreasing trend with age. The median value for 2 months-6 years was 4.77 (3.06,10.64), 6–10 years was 2.72 (1.14,7.03), 10–14 years was 1.55 (0.43,4.64) and 14–18 years was 0.88 (0.16,3.06) [41].

To date, the usage of NGAL as a predictor of AKI resolution was limited to very few studies in critically ill patients [28, 29, 42]. Srisawat et al., first described plasma NGAL as a useful biomarker for predicting renal recovery in AKI following community acquired pneumonia [42]. Following this, the BioMaRK study found that a decreasing uNGAL in the first 14 days was associated with greater odds of recovery in critically ill adults [27]. Furthermore, Moon et al., measured uNGAL every alternate day for 8 days in patients with AKI (*n* = 66) to determine AKI recovery which was defined as a 50% or greater decrease in plasma creatinine from the peak level. They found a good discrimination value for predicting AKI recovery [43]. Nickolas et al., in their single-center study have shown uNGAL to strongly predict sustained AKI with an AUC > 0.90 [44]. However, Meersch et al., showed contradictory findings; uNGAL in 50 adults post-cardiac surgery was a poor predictor for renal recovery at discharge [AUC (95%CI)- 0.48 (0.31–0.64)] [28]. Therefore, we believe that the evidence regarding the ability of uNGAL to differentiate persistent from transient AKI is equivocal.

The clinical settings in the above studies viz. sepsis, post-cardiac surgery in adults are high risk states for persistent AKI. On the contrary, in DKA, AKI mostly resolves. Also, the definitions of persistent AKI were quite variable. In this exploratory study, admission levels of uNGAL, uNCR, and the percentage of decline in both from admission to 24 h were found to be good predictors of persistent AKI in children with DKA. However, the positive predictive value (PPV) was low and negative predictive value (NPV) was high. Diagnostic accuracy, impact on patient-oriented outcomes and cost are the major determinants for any diagnostic test to be used in clinical practice; merely establishing a diagnosis may not translate to clinical benefit in all cases. Furthermore, given the low incidence of persistent AKI, routine use of uNGAL in a setting where majority of AKI resolves with hydration may be superfluous and not cost effective.

### Strengths and limitations

This is the first study of its kind in DKA that has explored the utility of uNGAL for persistent AKI in children. However, there are important limitations to this study. First of all, it is a post hoc analysis of a single-center study with relatively low sample size. Secondly, the population of children with DKA evaluated in this study was younger and more severely ill than is typical in other studies of DKA. Finally, this being a cross sectional analysis, long term risk of progression to CKD and its relation to uNGAL could not be fully explored. Future multicenter prospective studies may confirm the utility of uNGAL as early predictive biomarker for persistent AKI in DKA.

### Study implications

In our study, persistent AKI was characterized by lower decline in uNGAL during serial estimation. This is a novel finding and there are no similar reports in literature. It can help classify AKI in DKA as transient (responders to fluids) and persistent (those who will need additional targeted interventions like fluid titration, drug dose adjustment and RRT). Having said this, we believe that the majority of AKI in DKA is transient and resolves with hydration. The incidence of severe DKA and persistent AKI may be less in developed economies, where healthcare seeking is prompt unlike the developing economies. Despite this difference, recommending serial uNGAL measurement based on our findings may not be cost effective in resource limited settings.. Although both serum and urine NGAL may aid in risk stratification, its utility in prediction models need to be tested further in children.

### Conclusion

AKI at presentation is common in DKA. We found that uNGAL and uNCR had good correlation with serum creatinine and their elevation corresponded to the stage of AKI. While serial uNGAL measurement had good predictive ability for persistent AKI, uNCR performed better than uNGAL alone. Since most AKI resolved with hydration, the utility of uNGAL in predicting persistent AKI needs further evaluation in larger prospective studies.

## Supplementary Information

**Additional file 1.**

## Data Availability

The datasets used and/or analyzed during the current study are available from the corresponding author on reasonable request.
