# Supplementary material for: Serial urinary neutrophil gelatinase associated lipocalin in pediatric diabetic ketoacidosis with acute kidney injury
Source: Clin Diabetes Endocrinol. 2021 Nov 1;7:20. doi: 10.1186/s40842-021-00133-8 (PMC8559408; doi:10.1186/s40842-021-00133-8)
Supplement: Supplementary file 1 — Additional file 1. [file 40842_2021_133_MOESM1_ESM.docx]

**Additional file 1. Serial measurement of renal markers during first 48 hours**

|  | **No AKI** | **KDIGO Stage** | | |
| --- | --- | --- | --- | --- |
|  |  | **Stage 1** | **Stage 2** | **Stage 3** |
| **Number of children**, n (%) |  |  |  |  |
| Baseline | 19 (28.8) | 12 (18.2) | 24 (36.3) | 11 (16.7) |
| 24 hours | 48 (72.8) | 12 (18.2) | 3 (4.5) | 3 (4.5) |
| 48 hours | 57 (86.4) | 6 (90.1) | 1 (1.5) | 2 (3.0) |
| **Creatinine, mg/dl** |  |  |  |  |
| Baseline | 0.4 (0.14) | 0.66 (0.13) | 0.93 (0.2) | 1.49 (0.48) |
| 24 hours | 0.42 (0.12) | 0.6 (0.14) | 0.77 (0.3) | 2.26 (1.0) |
| 48 hours | 0.38 (0.1) | 0.56 (0.11) | 0.8 0.23) | 3.13 (0.47) |
| **GFR, ml/min** |  |  |  |  |
| Baseline | 96.4 (26.7) | 71.5 (4.8) | 49.6 (6.2) | 42.9 (10.9) |
| 24 hours | 117.8 (29.2) | 72.6 (5.4) | 55.5 (7.4) | 24.5 (12.1) |
| 48 hours | 130.2 (39.3) | 70.6 (6.4) | 52.9 (5.4) | 16.1 (3.5) |
| **uNGAL, ng/ml** |  |  |  |  |
| Baseline | 47.8 (21.9) | 65.4 (18.2) | 75.5 (24.6) | 88.6 (31.3) |
| 24 hours | 19.4 (14.7) | 23.1 (14.1) | 35.9 (1.8) | 86.8 (7.5) |
| **uNCR, ng/mg** |  |  |  |  |
| Baseline | 3.2 (1.6) | 5.5 (3.2) | 6.0 (3.7) | 8.1 (3.3) |
| 24 hours | 1.2 (1.0) | 1.1 (0.5) | 1.5 (1.0) | 11.1 (8.1) |

†All values expressed as Mean (Standard deviation)
